# Can Orthodontic Treatment Be Stable 20 Years after the End of the Treatment Scheme? Treatment of a Class 2, Division 1 Malocclusion with Severe Skeletal Discrepancy and Its 20-Year Follow-Up

**DOI:** 10.1155/2021/4810584

**Published:** 2021-09-30

**Authors:** Domenico Aiello, Riccardo Nucera, Stefania Costa, Michele Mario Figliuzzi, Sergio Paduano

**Affiliations:** ^1^Department of Health, University “Magna Graecia” of Catanzaro, Viale Europa, Loc. Germaneto, 88100 Catanzaro, Italy; ^2^Department of Biomedical and Dental Sciences and Morphofunctional Imaging, Section of Orthodontics, University of Messina, Italy

## Abstract

Class II malocclusions, after class I malocclusions, are the most frequent in the juvenile Italian population. They are most often skeletal in origin and due to mandibular retrusion. Functional devices seem to have a beneficial effect on the growth of the jaw. Long-term maintenance of the achieved results is essential for therapeutic success in any orthodontic treatment; moreover, the retention phase should last as long as possible, especially in the lower anterior sector. A female patient aged 10 years and 3 months presented a visibly convex profile and a severe mandibular retrusion. The anamnesis brought to light the habit of oral breathing and lower-lip sucking. The cephalometric analysis showed a normodivergent skeletal class II. The first treatment phase involved the use of a Bass type for 12 months at the end of the functional treatment; the second phase of fixed therapy was carried out following the principles of bioprogressive techniques. The photos at the end of treatment show an important improvement in the profile; a full class I ratio of molar and canine teeth was achieved with an excellent interarch relationship and a correction of the V-shaped upper arch. The result is occlusally and profilometrically stable after 2, 4, 5, 10, 14, and 20 years. The maintenance of a stable orthodontic result over time is the result not only of a correct and physiological occlusion but also and above all of a correct diagnosis and correct identification of problems that can cause the malocclusion itself. Flawed habits such as interposition of the lower lip and oral breathing must be intercepted and corrected early in order to correct them and not affect the long-term result of orthodontic treatment. In this case, a functional device associated with an orthodontic fixed finishing and a correct retention phase were necessary to correctly treat a second-class mandibular retrusion whose result remained stable 20 years after the end of therapy.

## 1. Case Report

Class II malocclusions are the second most frequent malocclusion after those in class I within the Italian population with an incidence from 32 to 40% [1, 2]. Different treatment options are available to clinicians. These range from simple fixed orthodontic treatment without extraction, through a two-phase treatment with functional appliances, to distalisation appliances, extractive fixed therapy, extractive therapy with orthodontic camouflage, or orthognathic surgery.

Since class II malocclusions are most often skeletal in origin and due to mandibular retrusion [3], the treatment of these defects is very frequently adequately successful through the use of fixed and mobile functional appliances, followed by a multibracket treatment. Treatment in one or two phases seems to produce no differences in terms of results, except for the risk of trauma to the incisors [4]. Functional devices, despite the considerable international debate, seem to have a beneficial effect on the growth of the jaw bone, by favouring an increase in its length in association with the dental effect [5] thus allowing good compensation and aesthetic improvement [6, 7]. The long-term maintenance of achieved results is essential for the therapeutic success of any orthodontic treatment [8]. Obviously, long-term stability is a multifactorial concept [9, 10]; it is obtained by achieving a correct occlusal relationship, following the concept of Andrews's six keys [11] associated with the correct movements of mandibular kinematics according to the concept of mutually protected occlusion [12, 13] and the correct balance of the muscular forces that could modify the result. Finally, a correct posttherapeutic retention phase cannot be ignored [14], with the aid of fixed or mobile retainers that avoid any possible orthodontic relapses. The retention phase should last as long as possible, especially for the lower anterior sector as it would seem to be the one most exposed to the risk of loss of obtained results over a long period from the end of the therapy, and to this end, the use of fixed retainers seems to find more indications than mobile ones in the long run [15].

With this article, we simply want to show how an orthodontic treatment, which reached ideal skeletal and occlusal parameters and was published previously in this journal, can remain stable over years.

### 1.1. Diagnosis and Aetiology

A female patient aged 10 years and three months presented a visibly convex profile and a severe mandibular retrusion. The anamnesis showed the habit of oral breathing and lower lip sucking.

**Figure 1:**
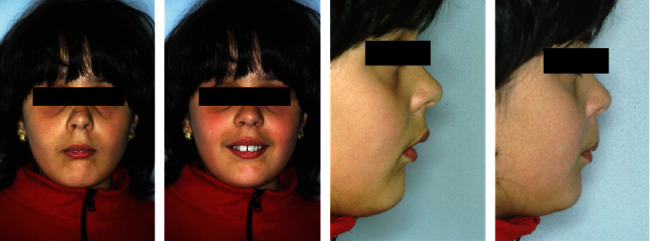
Initial extraoral photographs.

The extraoral photos (Figure 1) show a very convex profile, a soft tissue pogonion retrusion, lip incompetence due to an important sagittal discrepancy, and a very significant contraction of the orbicular muscle and the chin muscle when she was asked to close her lips. The habit of oral breathing was highlighted by the typical adenoid face with the presence of marked dark circles and a turned-up nose and small nostrils. Fraenkel's manoeuvre showed a noticeable improvement in the facial profile.

A functional examination showed that there were no lateral or anterior shifts. There were no signs or symptoms of joint problems. The lower lip when in its usual position was firmly held under the upper incisors.

**Figure 2:**
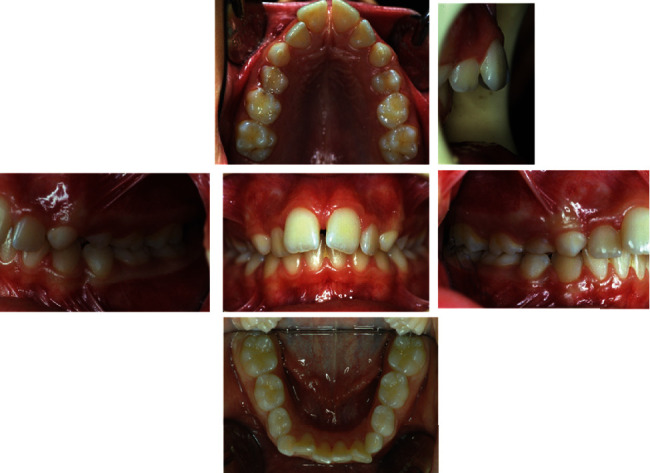
Initial intraoral photographs.

**Figure 3:**
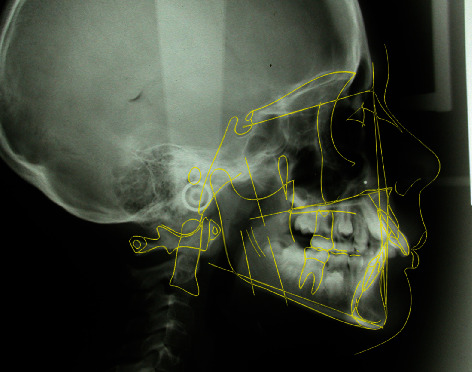
Initial lateral cephalogram.

The intraoral examination (Figure 2) and plaster models (Figure 3) showed a class II, division 1 malocclusion characterized by a marked overjet (12 mm) and overbite (4 mm). The upper arch had a V shape and rotation of elements 16 and 26 and full class II molar and canine relationship, with visible diastema between 11 and 21.

**Figure 4:**
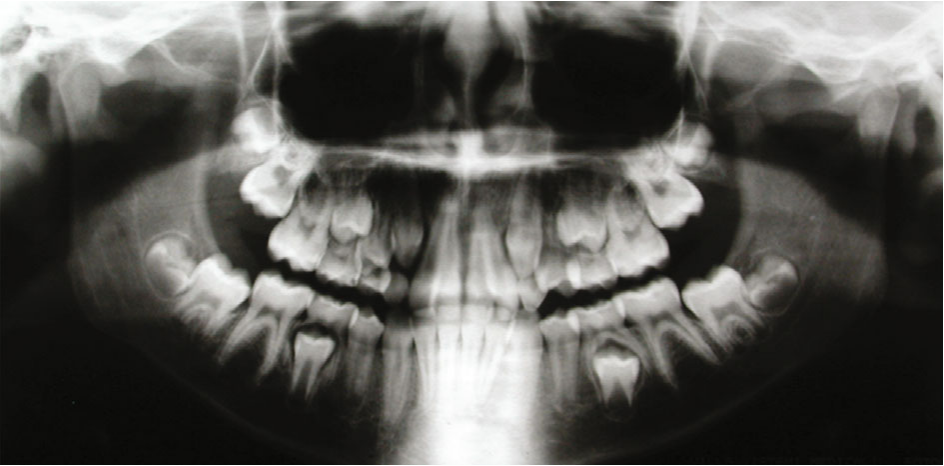
Initial panoramic radiograph.

The cephalometric analysis (Figure 4, Table 1) showed a skeletal class II (ANPg 5°) for mandibular retrusion (SNpg 78°). The patient had a normodivergent facial type (S-N/ANS − PNS = 4°, S-N/Go − Gn = 36°, Ans-Pns/Go − Gn = 31°). According to the analysis of the vertebral staging, the patient could be categorized in a type CS1 as there was no concavity lower than C2 and a trapezoidal shape of C3 and C4 and therefore at least one year earlier than the expected growth peak [16]. The panoramic X-ray showed a mixed dentition with reabsorption of the roots of the elements 53, 54, 55, 63, 65, 75, and 85, and the buds of the third molars in formation were evident.

### 1.2. Treatment Objectives

In order to conclude the treatment with the achievement of the results we set for ourselves, three possible therapeutic options were identified. The first alternative consisted in a six-monthly follow-up until the deciduous elements were completely exchanged. Once the final dentition was in place, a fixed orthodontic therapy could be carried out by extracting the first upper premolars in order to correct the overjet and the canine class. This type of therapy, however, was not considered as the mandibular retrusion would not have been corrected. This treatment would also lead to an inevitable deterioration of the profile with consequent overbite and apparent aging, associated with an increase in the difficulty in resolving the deep bite (4 mm of overbite.).

The second alternative was characterized by a six-monthly follow-up until reaching peak growth (CS3) in definitive dentition to allow the application of a fixed functional device such as the Herbst Miniscope, which would not only correct the skeletal and dental class but would also improve the patient's profile by perfectly achieving the treatment goals we requested.

Finally, the third therapeutic option was the use of mobile nociceptive functional equipment (Bass equipment) associated with a high extraoral traction in order to control the skeletal divergence and correct the sagittal relationship by mandibular advancement followed by fixed multibracket orthodontic therapy according to the principles of Professor Ricketts's bioprogressive techniques for the resolution of dental problems and a perfect finish and intercuspation in class I molars and canines.

The patient was advised to follow the third option because, as is well-explained in the systematic review by Thiruvenkatachari et al.; early treatment of second-class first division malocclusions is associated with a reduction in the risk of trauma to the incisors; furthermore, this process is advisable to reduce the risk of incisal trauma and to obtain the reduction of an increased overjet, whatever malocclusion the patient might present.

### 1.3. Treatment Progress

The patient underwent an orthodontic treatment in two phases.

The first phase involved the use of a Bass-type nociceptive mobile device. The patient wore this equipment for 20 h per day (following Malm and Green) for a period of 12 months to stimulate mandibular growth. High traction was associated with this type of equipment aiming to counteract mandibular postrotation and favouring a further improvement of the profile.

At the end of the functional treatment, a second phase of fixed therapy was carried out (Omniarch brackets Ricketts prescription 0.018^″^ × 0.030^″^; Dentsply GAC, 355 Knickerbocker Avenue Bohemia, NY 11716, United States) following the principles of bioprogressive techniques. The fixed therapy initially involved the use of a removable palatal bar associated with 2 sections (16-13 and 23-26) in order to expand the upper dental arch. Subsequently, the arches were levelled by using upper and lower arches 0.016 × 0.022 in nickel titanium (Rocky Mountains Orthodontics 650 W Colfax Ave Denver, Colorado, United States 80204). To favour the intrusion of the anterior sectors, the posterior sectors were then stabilized by means of yellow Elgiloy sectionals (Rocky Mountains Orthodontics 650 W Colfax Ave Denver, Colorado, United States 80204) above and below by associating 2 utility arches 0.016 × 0 022 of blue Elgiloy above and 0.016 × 0.016 blue Elgiloy below, while the retraction of the canines was possible with the aid of elastic chains. At the end of the therapy, yellow Elgiloy arches were used in order to correctly shape the arches and allow the finishing phases. The therapy lasted a total of 1 year and 8 months, and the result obtained was maintained using a 33-43 bonded retainer while no restraints were used in the upper arch.

## 2. Results of the Treatment

**Figure 5:**
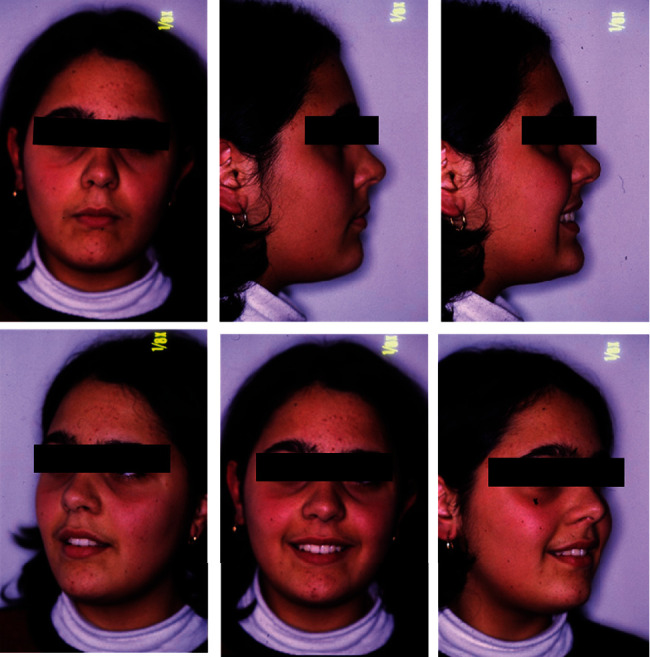
Final extraoral photographs.

**Figure 6:**
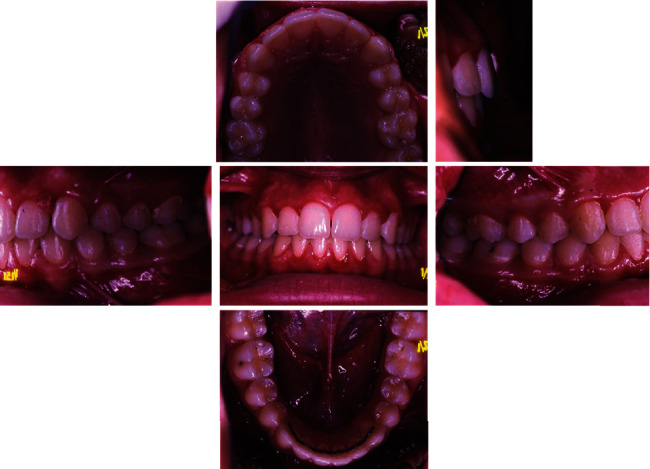
Final intraoral photographs.

**Figure 7:**
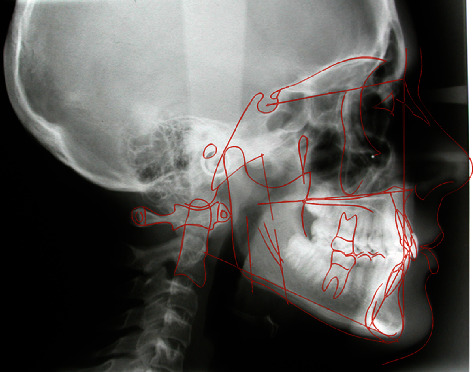
Final lateral cephalogram.

**Figure 8:**
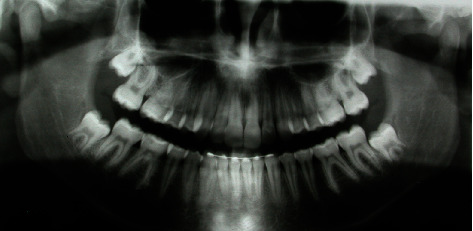
Final panoramic radiograph.

**Figure 9:**
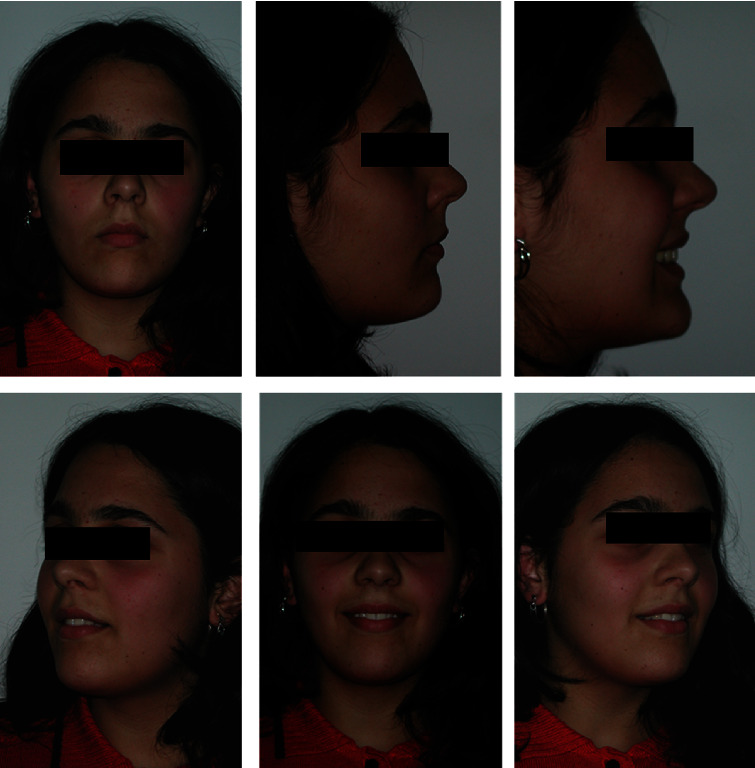
2-years follow-up extraoral photographs.

**Figure 10:**
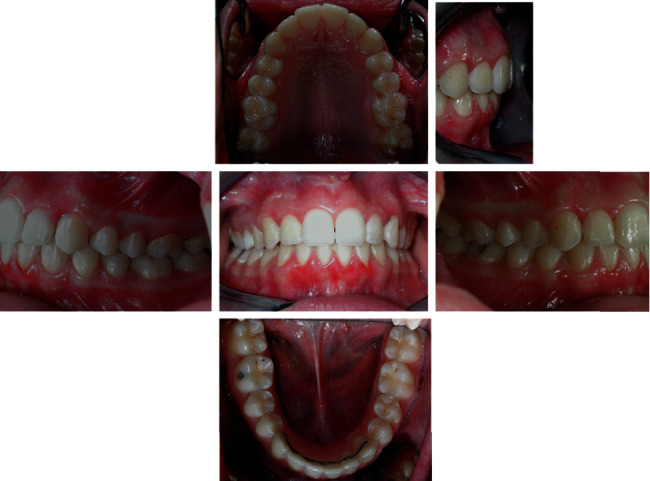
2-year follow-up intraoral photographs.

**Figure 11:**
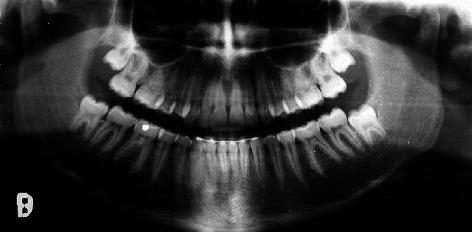
2-year follow-up panoramic radiograph.

**Figure 12:**
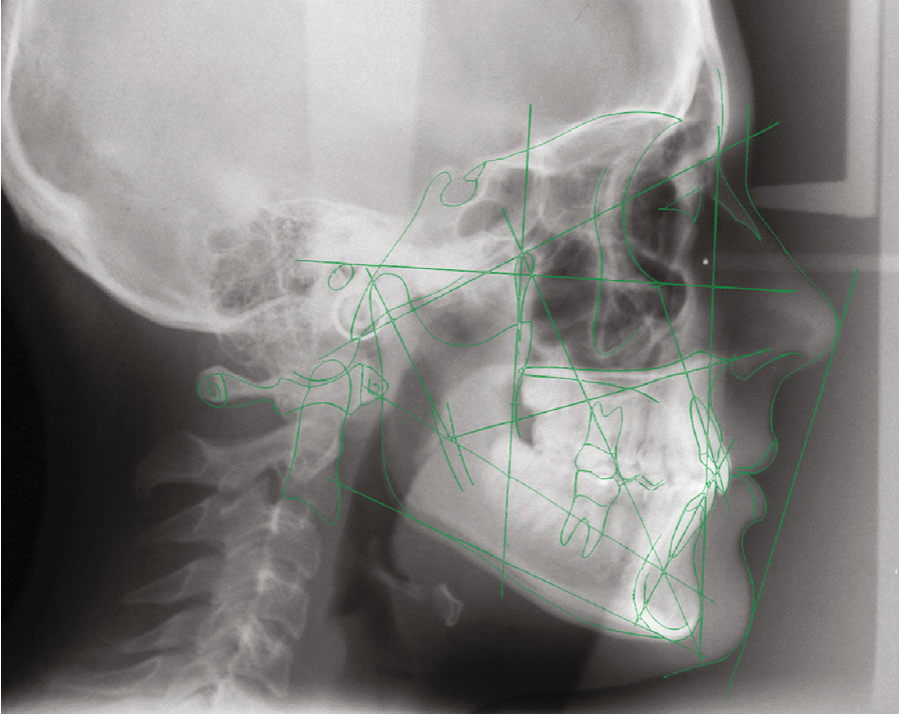
2-year follow-up lateral cephalogram.

**Figure 13:**
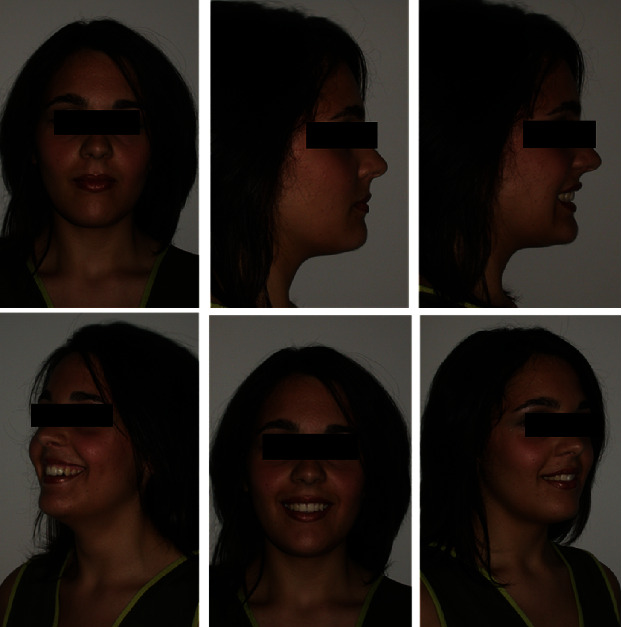
4-year follow-up extraoral photographs.

**Figure 14:**
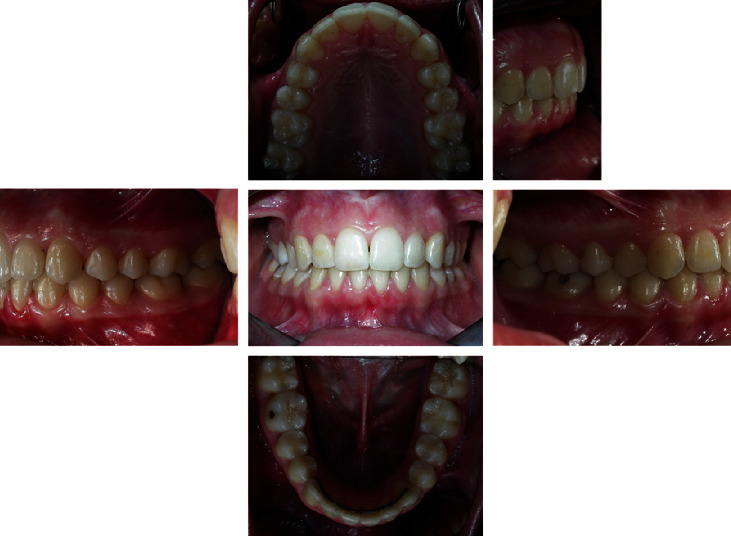
4-year follow-up intraoral photographs.

**Figure 15:**
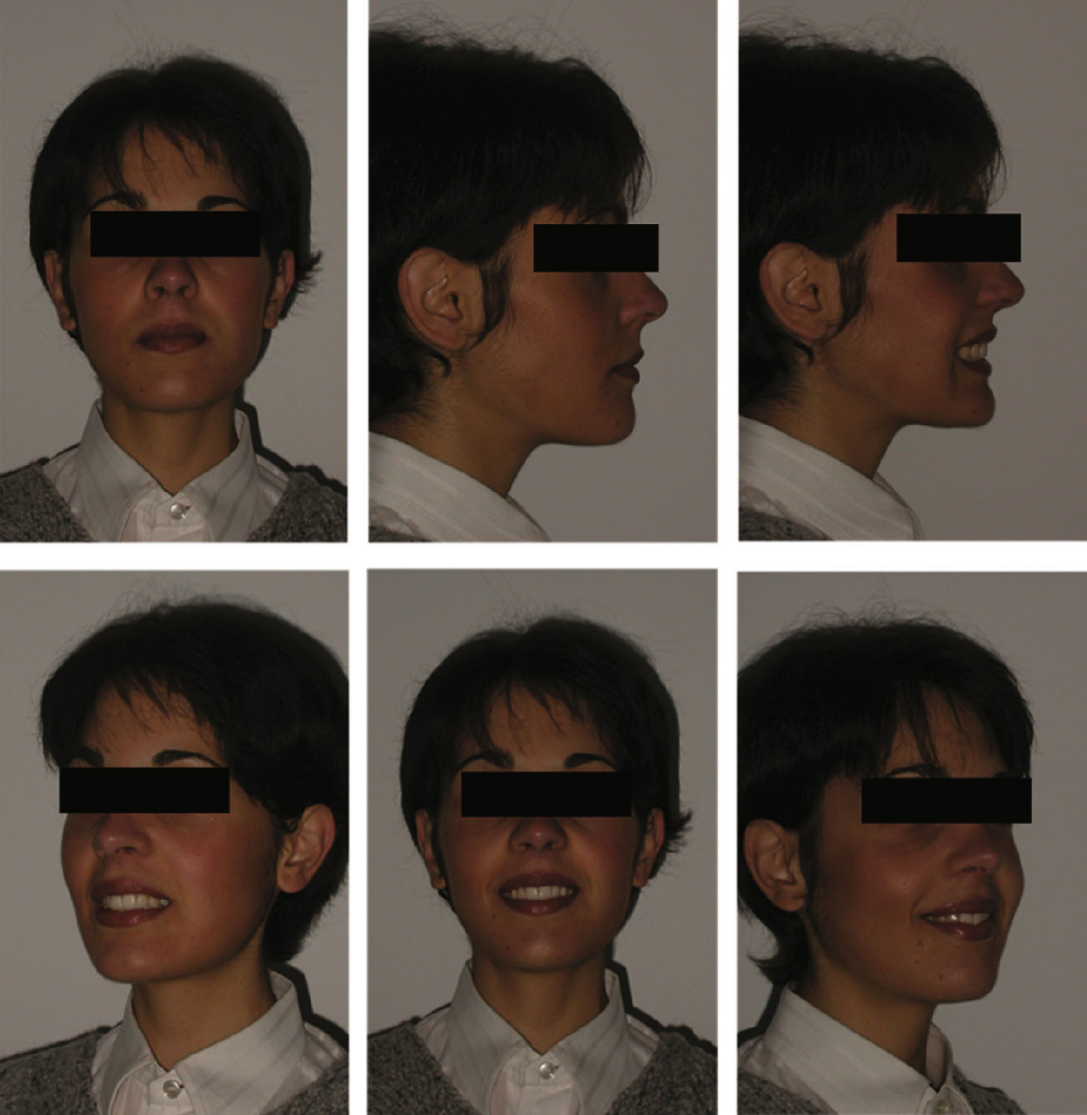
5-year follow-up extraoral photographs.

**Figure 16:**
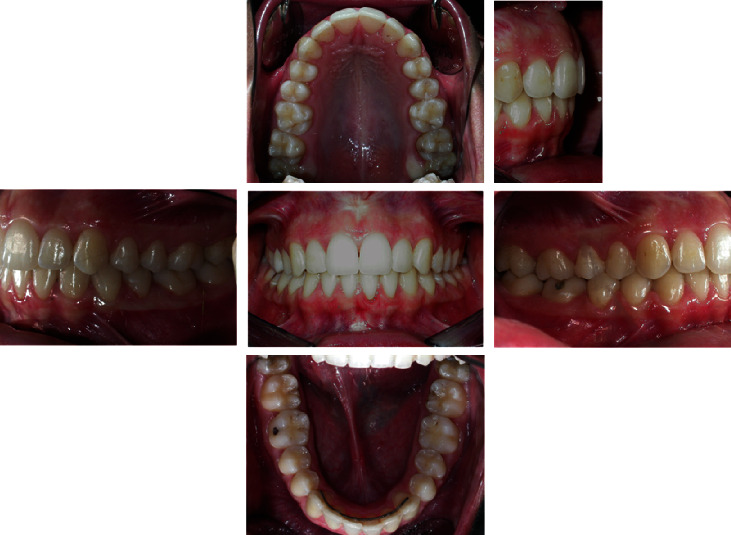
5-year follow-up intraoral photographs.

**Figure 17:**
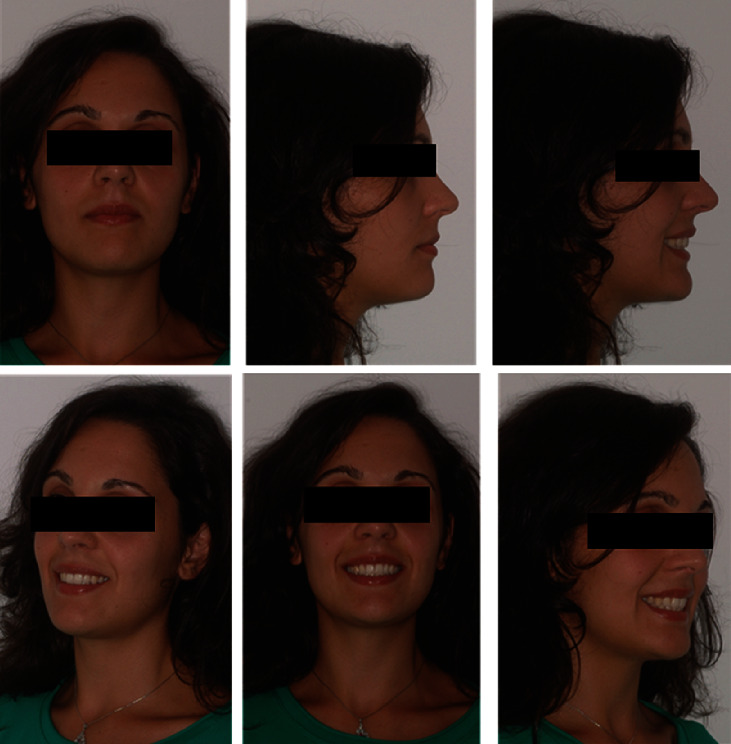
10-year follow-up extraoral photographs.

**Figure 18:**
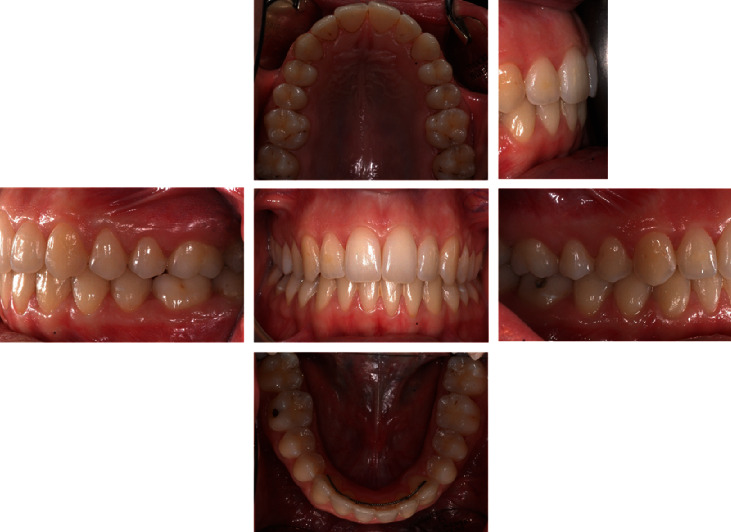
10-year follow-up intraoral photographs.

**Figure 19:**
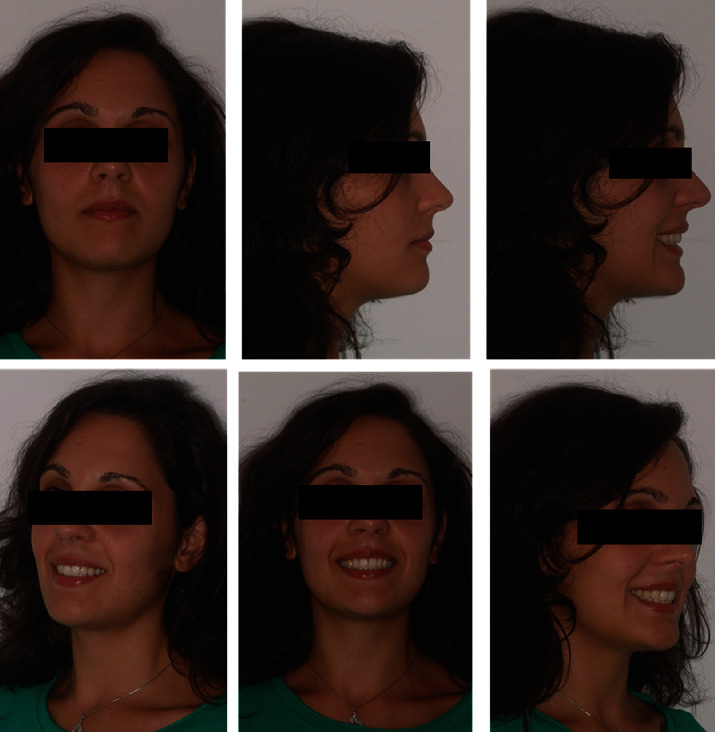
14-year follow-up extraoral photographs.

**Figure 20:**
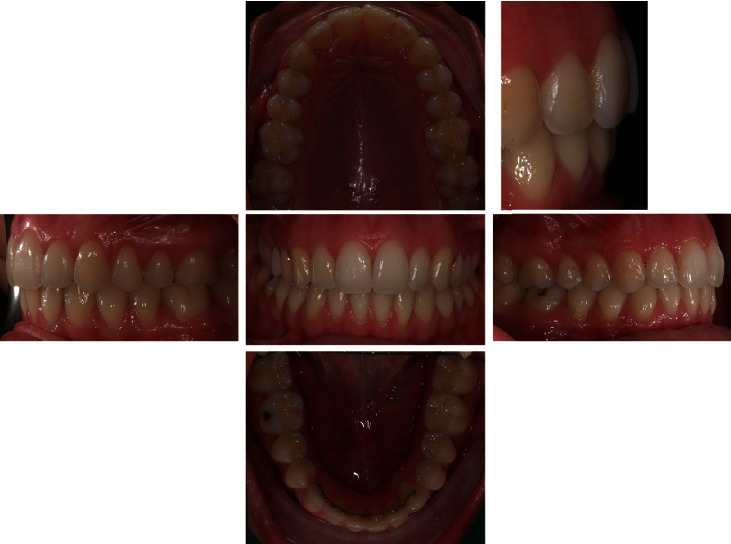
14-year follow-up intraoral photographs.

**Figure 21:**
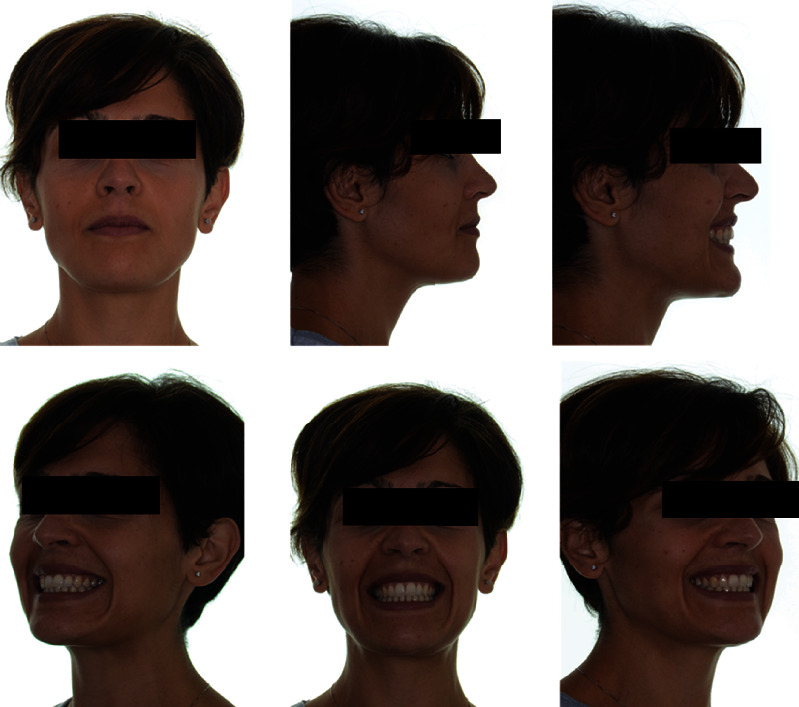
20-year follow-up extraoral photographs.

**Figure 22:**
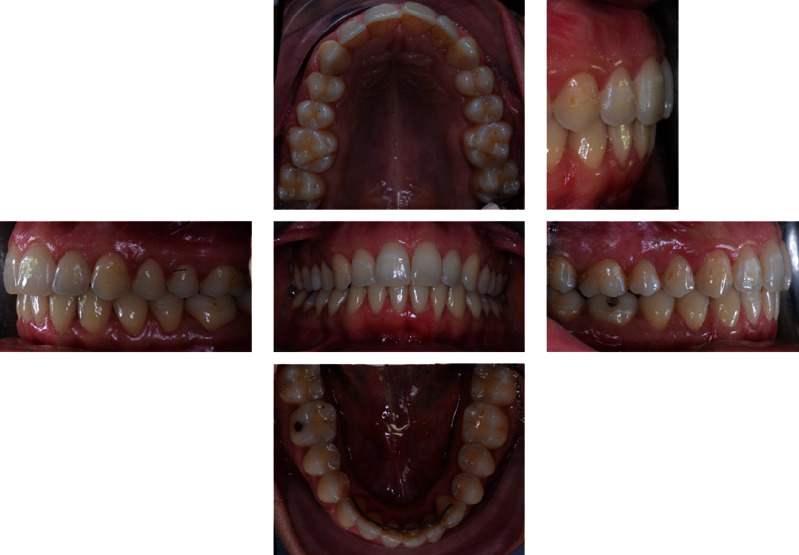
20-year follow-up intraoral photographs.

In the extraoral and intraoral photos (Figures 5 and 6), it is possible to see an important improvement in the profile, achievement of full lip competence without evident contractions of the orbicular and mental muscle. A full class I molar and canine relationship was achieved with an excellent interarch relationship and the correction of the V-shaped upper arch. Finally, complete correction of the overjet and overbite can be observed, measurable as 3 mm each (Figure 7, Table 2). The cephalometric analysis (Figure 8, Table 2) indicated a significant reduction of the ANPg angle, 0°, due to the correction of the skeletal discrepancy of the jaws, while the parameters that assessed the verticality were stable. The orthopantomography (Figure 9) showed correct root parallelism and the absence of reabsorption of the roots in the treated teeth. The functional analysis indicated the absence of pathological signs and symptoms affecting the temporomandibular joints and muscles. At the end of treatment, a fixed retainer 33-43 was placed bonded on all teeth for the lower jaw and a removable thermoformed retainer was placed in the upper jaw all lifelong that kept the treatment stable, as can be seen in Tables 3 and 4, after 2 years from the end of treatment. The result is occlusally and profilometrically stable after 2, 4, 5, 10, 14, and 20 years (Figures 10–22) as shown by the photos and radiographs taken.

## 3. Discussion

The maintenance of a stable orthodontic result over time is the result not only of a correct and physiological occlusion but also, and above all, of a correct diagnosis and correct identification of problems that can cause the malocclusion itself. Flawed habits such as interposition of the lower lip and oral breathing must be intercepted and corrected early in order to correct them and not affect the long-term result of orthodontic treatment [17, 18]. The correct diagnosis then passes to a scrupulous evaluation of the skeletal and aesthetic dental components [19, 20]. It is well known that skeletal malocclusions cannot be resolved solely at the dental level but necessarily require orthopaedic-functional treatment or even surgical orthodontic therapy. In the case of the second classes of mandibular retrusion, a functional therapy is undoubtedly the most used and studied over time [21, 22]. Functional devices such as Sander, Bass, Bionator, and Twin Block by Clark find their use mainly in this type of orthodontic therapy, through more or less similar systems. It is important, however, to keep in mind the therapeutic timing and, as previously expressed, the risk of trauma to the incisors in cases of patients with greatly increased overjets [23]. This condition is mostly associated with class II, division 1 malocclusion so that early treatment of this condition is often a duty on the part of the clinician, especially when the patients still have with mixed dentition [5, 24]. Finally, the scrupulous and careful finishing of the case is fundamental to the long-term therapeutic success [25, 26]. A case may be considered to have been treated adequately with the achievement of all 6 keys of occlusion. A very important role in maintaining the long-term result seems to be played by the number of occlusal contacts. These contacts must be reached during therapy as they are not formed during the posttreatment retention phase but rather tend in this phase to promote orthodontic recurrence if they are not positioned correctly [27]. Certainly, the fundamental characteristics of the mutually protected occlusion should not be underestimated, as these allow a reduction in the risk of the establishment of joint problems and avoid displacement forces in mandibular kinematic movements. Lastly, the type of device and the retention time involved in the postorthodontic phase are indispensable, especially for the lower anterior sector where the greatest probability of unwanted posttherapy movements occurs. The fixed-type restraints, even if they are more difficult to clean and are characterized by a decrease in comfort for the patient, are to be preferred for maintaining the obtained result. The retention time must be as long as possible [28] as even 10 years after the end of the therapy, the anteroinferior group is unstable [29]; the absence of restraint would therefore alter the orthodontic alignment achieved with consequent partial relapse of orthodontic treatment [30, 31].

## 4. Conclusions

A girl aged ten years and three months presenting a class II, division 1 malocclusion and a severe skeletal discrepancy as well as significant mandibular retrusion was correctly treated with the aid of Bass equipment followed by fixed therapy using bioprogressive techniques. The stimulation of mandibular growth associated with a correct finishing and dental gear was fundamental in order to improve the patient's profile and maintain the result obtained over time, even after 20 years from the end of the therapy.

## Figures and Tables

**Table 1:** Cephalometric values at the start of treatment.

*Sagittal skeletal relations*		
Maxillary position S-N-A	83.2°	82 ± 3.5°
Mandibular position S-N-Pg	78.1°	80 ± 3.5°
Sagittal jaw relation A-N-Pg	5.1°	2° ± 2.5°
*Vertical skeletal relations*		
Maxillary inclination S-N/ANS-PNS	4.1°	8 ± 3.0°
Mandibular inclination S-N/Go-Gn	36.3°	33 ± 2.5°
Vertical jaw relation ANS-PNS/Go-Gn	31.0°	25 ± 6.0°
*Dentobasal relations*		
Maxillary incisor inclination 1-ANS-PNS	109.2°	110° ± 6.0°
Mandibular incisor inclination 1-Go-Gn	95.2°	94° ± 7.0°
Mandibular incisor compensation 1-A-Pg (mm)	-2.1	2 ± 2.0
*Dental relations*		
Overjet (mm)	12.1	3.5 ± 2.5
Overbite (mm)	4.2	2 ± 2.5
Interincisal angle 1/1	122.1°	132° ± 6.0°

**Table 2:** Cephalometric values at the end of treatment.

*Sagittal skeletal relations*		
Maxillary position S-N-A	80.0°	82° ± 3.5°
Mandibular position S-N-Pg	80.1°	80° ± 3.5°
Sagittal jaw relation A-N-Pg	0.2°	2° ± 2.5°
*Vertical skeletal relations*		
Maxillary inclination S-N/ANS-PNS	6.2°	8° ± 3.0°
Mandibular inclination S-N/Go-Gn	34.0°	33 ± 2.5°
Vertical jaw relation ANS-PNS/Go-Gn	28.1°	25 ± 6.0°
*Dentobasal relations*		
Maxillary incisor inclination 1-ANS-PNS	105.0°	110 ± 6.0°
Mandibular incisor inclination 1-Go-Gn	95.0°	94° ± 7.0°
Mandibular incisor compensation 1-A-Pg (mm)	+2.2	2 ± 2.0
*Dental relations*		
Overjet (mm)	3.0	3.5 ± 2.5
Overbite (mm)	3.1	2 ± 2.5
Interincisal angle 1/1	133.0°	132° ± 6.0°

**Table 3:** Cephalometric values 2 years after the end of treatment.

*Sagittal skeletal relations*		
Maxillary position S-N-A	80.0°	82 ± 3.5°
Mandibular position S-N-Pg	80.2°	80 ± 3.5°
Sagittal jaw relation A-N-Pg	0.1°	2 ± 2.5°
*Vertical skeletal relations*		
Maxillary inclination S-N/ANS-PNS	6.3°	8 ±3.0°
Mandibular inclination S-N/Go-Gn	34.1°	33 ±2.5°
Vertical jaw relation ANS-PNS/Go-Gn	27.3°	25 ±6.0°
*Dentobasal relations*		
Maxillary incisor inclination 1-ANS-PNS	105.1°	110 ± 6.0°
Mandibular incisor inclination 1-Go-Gn	95.2°	94 ± 7.0°
Mandibular incisor compensation 1-A-Pg (mm)	+2.3	2 ± 2.0
*Dental relations*		
Overjet (mm)	3.0	3.5 ± 2.5
Overbite (mm)	3.1	2 ± 2.5
Interincisal angle 1/1	132.0°	132° ± 6.0°

**Table 4:** Comparison of cephalometric values: before, after, and 2 years posttherapy.

	Initial	Final	Follow-up	
*Sagittal skeletal relations*				
Maxillary position S-N-A	83.2°	80.0°	80.0°	82 ± 3.5°
Mandibular position S-N-Pg	78.1°	80.1°	80.2°	80 ± 3.5°
Sagittal jaw relation A-N-Pg	5.1°	0.2°	0.1°	2 ± 2.5°
*Vertical skeletal relations*				
Maxillary inclination S-N/ANS-PNS	4.1°	6.2°	6.3°	8 ± 3.0°
Mandibular inclination S-N/Go-Gn	36.3°	34.0°	34.1°	33 ± 2.5°
Vertical jaw relation ANS-PNS/Go-Gn	31.0°	28.1°	27.3°	25 ± 6.0°
*Dentobasal relations*				
Maxillary incisor inclination 1-ANS-PNS	109.2°	105.0°	105.1°	110 ± 6.0°
Mandibular incisor inclination 1-Go-Gn	95.2°	95.0°	95.2°	94 ± 7.0°
Mandibular incisor compensation 1-A-Pg (mm)	-2.1	+2.2	+2.3	2 ± 2.0
*Dental relations*				
Overjet (mm)	12.1	3.0	3.0	3.5 ± 2.5
Overbite (mm)	4.2	3.1	3.1	2 ± 2.5
Interincisal angle 1/1	122.1°	133.0°	132.0°	132 ± 6.0°
